# Environmental Determinants of Pediatric Obesity: An Epidemiological Review

**DOI:** 10.3390/epidemiologia7020036

**Published:** 2026-03-02

**Authors:** Doha Hassan, Mostafa Salama, Reham Ahmed, Seema Kumar

**Affiliations:** 1 Department of Pediatric and Adolescent Medicine, Mayo Clinic, Rochester, MN 55905, USA; hassan.doha@mayo.edu (D.H.); salama.mostafa@mayo.edu (M.S.); 2 Division of Pediatric Endocrinology, Department of Pediatric and Adolescent Medicine, Mayo Clinic, Rochester, MN 55905, USA; 3 Department of Pathology, Mayo Clinic, Rochester, MN 55905, USA; ahmed.reham@mayo.edu

**Keywords:** children, obesity, overweight, environmental, built environment, screen, obesogens

## Abstract

Pediatric obesity represents an urgent public health concern, with rapidly increasing prevalence across all regions. While genetic susceptibility contributes significantly to interindividual variability in weight, the significant increase in obesity prevalence over the last 30 years is driven by shifts in environmental contributors. This narrative review will summarize evidence on the major environmental determinants of childhood obesity. Environmental contributors to obesity include the food environment, physical activity and built environments, socioeconomic and community context, home and family environments, digital exposures, early life and chemical obesogens and policy drivers. These environmental factors influence activity patterns, dietary habits, sleep, and stress. Additionally, many of these contributing factors cluster within communities that are disadvantaged, thereby increasing predisposition of specific racial, ethnic and socioeconomic groups to childhood obesity. We highlight research gaps and opportunities for multisectoral interventions aligned to impact the growing prevalence of childhood obesity.

## 1. Introduction

The prevalence of obesity among the pediatric population has assumed epidemic proportions [1]. A recent global meta-analysis pooling data from 2033 studies across 154 different countries, including 45.9 million children and adolescents, estimated the worldwide obesity prevalence at 8.5% and of overweight at 14.8% with marked geographic disparities [2]. Childhood obesity is associated with substantial immediate and long-term health outcomes, including hypertension, dyslipidemia, type 2 diabetes, and metabolic dysfunction-associated steatotic liver disease [3,4]. These conditions frequently persist into adulthood and are linked to higher risks of premature cardiovascular disease, stroke, and mortality [3,5].

Genetic factors are estimated to account for 40–75% of the interindividual variability in body mass index (BMI) [6,7]. Numerous studies demonstrate that the heritability of BMI varies according to environmental context [8] and that risk alleles (e.g., *FTO*, *MC4R*) exert stronger or weaker effects depending on physical activity levels, dietary environment, and socioeconomic conditions [9,10]. Additionally, early-life epigenetic mechanisms represent a key pathway through which environmental exposures modulate genetic susceptibility [11].

**Figure 1:**
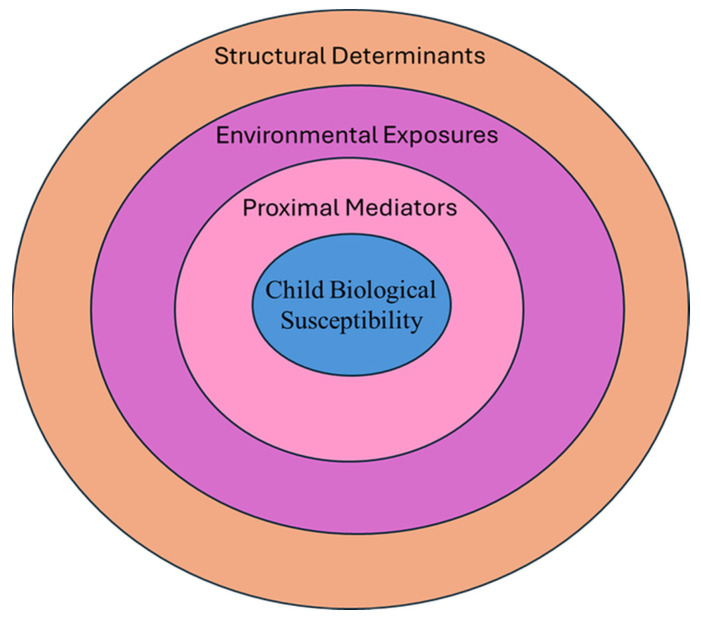
Life-course socioecological framework of pediatric obesity. This figure illustrates a layered, life-course model in which pediatric obesity risk emerges from interactions across multiple levels of influence. The outer layer represents upstream structural determinants, including socioeconomic status, racism and structural inequities, and policy context. The middle layer captures environmental exposures such as the food and built environments, digital media, and chemical exposures. The inner layer reflects proximal mediators—diet quality, physical activity, sleep, psychosocial stress, and the gut microbiome. At the core, individual biological susceptibility, shaped by genetics, epigenetic programming within the Developmental Origins of Health and Disease (DOHaD) framework, and neuroendocrine regulation, modifies vulnerability across developmental stages.

Existing reviews of pediatric obesity determinants often focus on single domains—such as diet, physical activity, or chemical exposures—without integrating social, built, and early-life environments within a unified conceptual framework [12,13,14]. Moreover, many prior syntheses are geographically concentrated in high-income countries and do not adequately capture the data from low- and middle-income regions undergoing rapid nutrition and urban transitions [15,16,17,18]. This narrative review is grounded in a life-course and socioecological framework in which structural and social determinants shape interconnected environmental exposures that influence childhood obesity risk (Figure 1). The aims of this review are to (1) analyze epidemiological evidence from diverse studies across the globe, (2) identify areas of consistent versus limited or conflicting evidence, and (3) delineate key research gaps to inform prevention and policy efforts.

## 2. Methods

This review was conducted as a narrative epidemiological synthesis and incorporated structured methodological elements to enhance rigor and transparency. Given its narrative design, the objective was to identify and synthesize high-quality and influential evidence across multiple environmental domains, rather than to exhaustively capture all available studies. An extensive literature review was performed using PubMed, Web of Science, and Google Scholar for articles published between January 1990 and January 2025, without geographic restrictions. Keywords and Medical Subject Headings (MeSH) terms linked to pediatric obesity and environmental exposures were used, involving (“Pediatric Obesity” OR childhood obesity OR pediatric overweight) AND (“Environment” OR environmental exposure OR built environment OR food environment OR endocrine disruptors OR air pollution OR screen time OR socioeconomic) AND (“Epidemiology” OR epidemiology OR prevalence OR risk). Full search strategies are provided in Appendix A. Eligible studies included original scholarly articles, systematic reviews, and meta-analyses examining environmental, social, or early-life determinants of pediatric obesity. Inclusion criteria were: (1) study populations of children and adolescents; (2) a primary focus on environmental determinants such as food and built environments, socioeconomic and community context, digital exposures, early-life factors, or chemical exposures; and (3) publication in English. Exclusion criteria included non–peer-reviewed material, adult-only studies, studies focused exclusively on genetic determinants, and publication types such as letters, case reports, commentaries, book chapters, and conference abstracts. Consistent with the narrative scope of the review, a focused set of approximately 400 records was identified and screened for eligibility. Stages of article screening included title and abstract screening followed by full-text review, conducted independently by four reviewers (D.H., M.S., R.A., and S.K.). Final decisions were made by D.H. and S.K. through consensus review, with discrepancies resolved through discussion. After application of inclusion and exclusion criteria, 152 studies were selected for the final synthesis.

## 3. Food Environment

The food environment is one of the leading environmental contributors to childhood obesity, as summarized in Table 1 and Table 2. The following sections outline the association between key components of the food environment and pediatric obesity.

### 3.1. Ultra-Processed Foods

High intake of ultra-processed foods (UPF) is consistently linked to a higher risk of childhood obesity and excess adiposity [19,47,48]. UPF tend to be calorie-rich with unbalanced nutritional profiles and promote excessive energy intake through their high calorie content, enhanced palatability, and soft consistency, which can disrupt satiety signaling and food-reward pathways [14,19]. UPF currently accounts for approximately 2/3 of total energy consumption in the U.S. pediatric population [61]. Longitudinal cohort data from the US, UK, and other global settings indicate that increased UPF intake in children is associated with a higher annual surge in BMI, fat mass, and waist circumference, with several studies demonstrating a dose–response relationship [48,62,63]. In the U.S. Growing Up Today Study, each 10% rise in UPF consumption was linked to a yearly BMI rise of 0.01 (95% confidence interval (CI): 0.003–0.03) and a cumulative increase of 0.07 (95% CI: 0.01–0.13) over five years. Similarly, in the Pelotas 2004 Birth Cohort from Brazil, an increase of 100 g/day in UPF intake was linked to a 0.14 kg/m^2^ increase in the fat mass index (FMI) [62,63]. However, effect sizes are modest, and estimates may be influenced by residual confounding from SES, overall diet quality, and correlated lifestyle behaviors. In addition, variation in UPF classification systems and dietary assessment methods limits comparability across studies. In a systematic review by Petridi et al., including 17 observational studies of children and adolescents (≤18 years), positive associations between UPF consumption and overweight/obesity or cardiometabolic risk were found. However, four studies (three cross-sectional and one cohort) reported null associations. Notably, studies demonstrating positive associations were predominantly longitudinal with longer follow-up, whereas null findings were largely derived from cross-sectional designs, which are limited in their ability to capture cumulative effects of sustained UPF exposure over time [64]. Direct evidence from intervention studies specifically aimed at reducing childhood obesity by decreasing UPF intake is scarce. 

Multiple systematic reviews and meta-analyses of cohort studies and randomized clinical trials demonstrate a positive, dose-dependent relationship between SSB consumption and increased weight in children [49,50,65]. Randomized trials in school-based interventions replacing SSB with non-caloric beverages or water or providing education and environmental changes have demonstrated a lower increase in BMI and reduced odds of overweight/obesity, particularly among children at higher baseline risk [66]. The overweight risk was reduced by 31% (*p* = 0.04) in one study that included both provision of water and education [67]. Notably, many of the trials primarily demonstrate short- to medium-term effects on beverage intake and weight trajectories, with limited evidence of long-term BMI or obesity outcomes beyond the intervention period. In a cross-sectional study conducted in secondary schools in three cities in Saudi Arabia, a negative relationship between SSB consumption and obesity was found. Lower consumption of SSB (<3 days/week) was linked to increased odds of overweight/obesity (adjusted odds ratio (aOR = 1.32, 95% CI: 1.08–1.62) [68]. This counterintuitive finding suggests that adolescents with obesity in this Saudi cohort reported lower SSB consumption, potentially reflecting reverse causality (i.e., dietary modification following weight gain), reporting bias, or incomplete capture of overall dietary quality. Cultural practices, lifestyle factors, and socioeconomic context—none of which were fully accounted for—may also have confounded the observed association.

Higher intake of dietary fiber, fruits, and vegetables is linked to a lower odds of childhood obesity [13,69,70,71]. Observational and cohort studies consistently show inverse associations between fruit, vegetable, and fiber intake and measures of adiposity in children, though the effect sizes are small [72]. Intervention studies and meta- analyses indicate that programs aimed at increasing fruit and vegetable intake can improve dietary behaviors and may reduce obesity prevalence, but the impact on BMI is limited [13,73,74]. For instance, a meta-analysis of fruit intake interventions in children found a significant increase in fruit consumption and a reduction in obesity prevalence (OR = 0.74, 95% CI: −1.15 to −0.05 cm, *p* < 0.06) but only a small, non-significant reduction in BMI (mean difference−0.27 kg/m^2^ and a modest decrease in waist circumference [72]. These findings highlight a common pattern across food-based interventions: improvements in dietary behaviors often precede, and do not always translate into, sustained changes in weight status.

### 3.2. Food Deserts and Food Swamps

Several cross-sectional and cohort studies demonstrate that children living in neighborhoods with decreased income and access to food (food deserts) have higher BMI and higher odds of obesity from childhood to adolescence [51,75,76]. Similarly, exposure to food swamps (areas in outlets with higher access to unhealthy foods) in both residential and school environments is linked to increased odds of being overweight in the pediatric population [13,77]. However, definitions of food deserts and food swamps vary substantially across studies, and disentangling their independent effects from broader neighborhood socioeconomic conditions remains challenging. In a recent pooled analysis of over 100 studies, closer proximity or greater density of fast food outlets was remarkably linked to increased odds of obesity (OR = 1.15; 95% CI: 1.02–1.30), whereas greater access to supermarkets was linked to lower obesity risk (OR ≈ 0.90; 95% CI: 0.82–0.98). Availability of fresh fruit and vegetable outlets was also negatively linked to obesity (OR ≈ 0.93; 95% CI: 0.90–0.96) [78]. These findings reflect nuanced relationships between the food environment and obesity and support the conceptualization of unhealthy retail environments as obesogenic contexts. A systematic review concluded that increasing access to and proximity of healthy food outlets can improve dietary quality and odds of obesity [79]. Additionally, increased exposure to convenience stores raises children’s BMI, while increased access to small grocery stores selling healthy items lowers BMI in children [80]. Overall, observed associations are modest and context-dependent, and residual confounding by neighborhood characteristics, SES and cultural factors cannot be fully excluded.

### 3.3. Food Marketing and Advertising

Food marketing and advertising influence children’s eating behavior. Exposure to food marketing, especially for fat-, sugar- and salt-rich food, leads to increased food intake, stronger preference for unhealthy foods and more frequent purchase requests among children and adolescents [20,81,82]. These effects are observed across various media, including television and digital platforms, and are particularly pronounced in younger children due to their developmental vulnerability [20,21]. Real-world policy interventions, such as advertising bans in Quebec and the UK, have shown a decline in children’s access to unhealthy food marketing and favorable trends in dietary behavior [83]. Importantly, much of the existing evidence demonstrates short-term effects on preferences and intake, while evidence linking marketing exposure to sustained changes in BMI or obesity risk remains limited. Online advertising leverages algorithms, personalized recommendations and embedded advertisements in streaming platforms to target children with unhealthy food promotions [38,39].

### 3.4. School and Childcare Nutrition Environments

Schools make a significant contribution to framing children’s lifestyle behaviors. School-based interventions, particularly those that modify the food environment (healthier cafeteria offerings, limiting access to sugary drinks), increase physical activity and involve family or community components, can lead to small but meaningful reductions in BMI and improvement in dietary behaviors [22,23]. A meta- analysis found that school food environment interventions reduced BMI z-scores (standard mean difference −0.12, 95% CI: 0.10–0.15) and increased fruit intake [22]. However, most studies report modest effect sizes, and sustained impacts on weight status beyond the intervention period are less consistently observed. Interventions in childcare centers, especially those that are longer in duration, multicomponent (targeting both nutrition and physical activity) and involve parents, can improve dietary behaviors and, to a lesser extent, reduce obesity risk [84,85,86]. A cluster- randomized trial showed that a childcare-based intervention improved the nutrition environment and promoted healthy weight, though changes in physical activity were less pronounced [85]. In general, significant effects on weight status are less consistently noted than in school-based studies. Food environment exposures frequently co-occur with socioeconomic disadvantage, neighborhood deprivation, and targeted digital marketing, highlighting the interdependence of dietary, social, and media environments in shaping obesity risk [51,83].

## 4. Built Environment and Physical Activity

The built environment—comprising neighborhood layout, land use, transportation systems, and access to green space—plays a central role in shaping opportunities for physical activity. In systematic reviews and meta-analyses of longitudinal studies, neighborhoods with greater walkability and access to green spaces and parks are consistently associated with decreased BMI and decreased odds of overweight/obesity in children, likely by promoting physical activity, discouraging sedentary behaviors, and providing safe recreational spaces [13,17,24,25]. However, observed associations vary across studies in part due to substantial methodological heterogeneity, including differences in how built environment exposures are measured (e.g., Geographic Information Systems (GIS)-based indicators, parental perceptions, field audits, or satellite-derived greenness indices). In an analysis of data from the National Survey of Children’s Health (NSCH), the risk of overweight or obesity was greater with the declining number of sidewalks, parks, playgrounds, and recreation centers and increasing number of abandoned buildings and evidence of vandalism or crime), with the greatest odds seen with no amenities and all three detractions (OR = 1.71; 95% CI: 1.3–2.11) [26].

In a retrospective multi-cohort observational study in 20,677 children in the Child Health Outcomes (ECHO) program across the US, two composite indices were used: the Child Opportunity Index (COI) and the Social Vulnerability Index (SVI) [87]. The COI incorporates measures such as median household income, availability of healthy food options and green spaces, a walk-friendly environment, and exposure to environmental toxins. In contrast, the SVI assesses neighborhood disadvantage and vulnerability based on 15 social factors, including SES and household structure. Children living in areas with greater COI scores had decreased mean BMI and a reduced likelihood of obesity across follow-up compared with those in areas with very low COI, with a mean BMI difference of β = −2.58 (95% CI: −2.95–−2.21). Similarly, children residing in neighborhoods with lower SVI scores exhibited remarkably decreased mean BMI and decreased risk of obesity longitudinally compared with peers in areas characterized by high social vulnerability [87]. While such composite indices capture multidimensional neighborhood context, they may obscure the relative contributions of individual built environment components and complicate causal interpretation. In a systematic review and meta-analysis of 1104 children with a median follow-up of 3.5 years, living in unsafe neighborhoods was associated with a decline in children’s physical activity by 0.13 h/week and an increase in BMI [24]. A cross-sectional ecological study in Lisbon observed that children residing in newer, greener neighborhoods were less likely to have obesity [88]. Nonetheless, cross-sectional designs and ecological analyses are at risk of selective residential choice, reverse causality, and confounding.

Built environment features that enhance walkability, in particular, appear to be protective against pediatric obesity. In a large US longitudinal observational cohort study, greater increases in intersection density over nine years (1998–2007) were linked to 21% decreased odds of obesity (OR = 0.79; 95% CI, 0.66–0.94) [52]. Higher residential density was also protective, predicting lower odds of obesity (OR = 0.54; 95% CI: 0.30–0.98) and overweight (OR = 0.54; 95% CI, 0.30–0.95) [52]. In a prospective cohort study from an urban pediatric health system spanning 2007 to 2016 (*n* = 51,873, ages 6–19 years, 77% African American), an increase in green spaces and safetyof neighborhood were associated with reductions in BMI z-scores (mean change in BMI z-score per 1 SD increase for both variables: −0.012; 95% CI: −0.018 to −0.007) [25]. Although these results indicate potential dose–response relationships, effect sizes are small, and residual confounding related to socioeconomic conditions and family-level behaviors cannot be fully excluded.

On a global level, a study conducted in the UAE indicated that parental perceptions of micro-environment safety (e.g., sidewalks, traffic conditions, and neighborhood appeal) influence children’s physical activity and, by extension, weight-related outcomes, illustrating how socio-cultural and environmental contexts in the Middle East shape obesity risk [17]. In a longitudinal analysis of 9589 Australian children followed from early childhood through adolescence, favorable neighborhood characteristics were associated with healthier BMI trajectories. Children living in neighborhoods perceived as very safe had lower risks of extreme BMI increase (adjusted relative risk ratio RRR 0.71, 95% CI 0.62–0.82), while improvements in green space quality over time were similarly protective (RRR 0.60, 95% CI 0.43–0.84). In contrast, greater access to shopping facilities, particularly increasing or consistently high access, was associated with higher risks of extreme BMI gain (RRRs 1.46–1.64). High and persistent traffic exposure was also associated with increased BMI trajectories (RRR 1.35, 95% CI 1.11–1.64) [27]. Collectively, these findings suggest that environmental influences on pediatric obesity are globally relevant but exhibit substantial contextual variation in exposure patterns, cultural norms, and policy environments.

In multiple longitudinal and cross-sectional observational studies, higher crime rates and lower perceived safety are related to increased risk of obesity, likely due to reduced outdoor activity [25,26,27,28]. A systematic review and meta-analysis of 22 prospective studies conducted in seven countries concluded that residing in unsafe neighborhoods was linked to a reduction in children’s physical activity by 0.13 h/week [24]. Notably, perceptions of safety may differ from objectively measured crime rates, further contributing to heterogeneity in observed associations.

In a non-randomized controlled trial (2003–2005) using community-based interventions in three diverse cities in Massachusetts, one with an intervention aim to increase physical activity and availability of healthful foods and two socio-demographically matched control communities, the BMI z-score of children in the intervention group declined by −0.06 [*p* = 0.005, 95% CI: −0.08 to −0.04] compared to controls [89]. However, infrastructural interventions are challenging to evaluate experimentally, often lack randomization, and may be influenced by concurrent social or policy changes, limiting causal attribution.

Taken together, evidence from multiple study designs—including longitudinal cohorts, pooled multi-cohort analyses, systematic reviews, and quasi-experimental interventions—demonstrates a consistent, dose-responsive association between favorable built environment characteristics and lower childhood obesity risk, supporting a robust and reproducible relationship despite the observational nature of much of the literature. Nevertheless, heterogeneity in exposure measurement, modest effect sizes, and limitations in causal inference warrant cautious interpretation of these findings.

## 5. Socioeconomic and Community Context

Lower SES and disadvantaged community contexts are related to greater risk of obesity in the pediatric population, with both individual, family-level, and neighborhood-level factors contributing independently and interactively [90,91,92]. These determinants can be conceptualized as upstream structural factors—such as household income, parental education, occupation, and neighborhood deprivation—that shape downstream behavioral and biological exposures across the life course (Figure 1). Importantly, most evidence linking SES to childhood obesity is observational, and associations are subject to residual confounding by correlated social, environmental, and family-level factors. The underlying mechanisms include poor access to healthy foods, decreased options for physical activity, heightened stress and adverse childhood experiences. Community factors such as neighborhood food access, school environment and safety also play a critical role.

Parental education and occupation are important SES indicators, with low parental education consistently linked to higher pediatric obesity prevalence in high-income countries, while the direction and magnitude of these associations are more variable in middle-income settings, reflecting differences in nutrition transitions, food systems, and social gradients of obesity [92]. In a multi-cohort analysis including 26,565 children from six high-income countries, lower maternal education and household income were associated with greater obesity risk at ages 8–11 years [53]. Children with low maternal education had nearly a threefold greater obesity risk (pooled RR = 2.99), while those from lower-income households had over twice the risk (RR = 2.69) [53]. Data from a U.S. national birth cohort of 1211 mother–child dyads also showed that children from families with lower maternal education or income were significantly more likely to develop obesity [93]. A substantial proportion of this socioeconomic gradient is mediated through modifiable prenatal and early-life pathways rather than being fully explained by structural SES alone [93]. Mediating pathways include higher maternal preconception BMI, smoking during pregnancy, accelerated infant weight gain, shorter sleep duration, increased screen time, and less cognitively and emotionally enriching home environments [93].

Food insecurity and limited access to fresh, healthy foods can drive families toward cheaper, calorie-dense options, increasing obesity risk [5,32]. In a three-year prospective study of 534 parent–child dyads, early-life food insecurity was linked to higher BMI z-scores in children aged 2–4 years, with elevated BMI persisting through follow-up. Dietary quality did not mediate this relationship [94]. This suggests that food insecurity may influence obesity risk through stress-related, behavioral, or metabolic pathways beyond nutrient composition alone. In a review of NHANES data from 1962, children aged 6–18 years, more than one-quarter of children lived in food-insecure households, and these individuals had greater odds of both obesity (aOR = 1.59, 95% CI: 1.19–2.13) and abdominal obesity (aOR = 1.56, 95% CI: 1.19–2.03) [54]. Food insecurity disproportionately affects racial and ethnic minority populations, reflecting the intersection of SES, structural disadvantage, and cultural context in shaping obesity risk [54].

The most effective community-level interventions for reducing childhood obesity in low SES and disadvantaged neighborhoods are multi-level, multi-component programs that address both structural constraints and individual-level mediating behaviors. Programs that simultaneously target school, family, and community environments—such as the Stanford GOALS trial—have demonstrated remarkable decline in BMI trajectory and improvements in cardiometabolic risk factors over 1–2 years [95]. These interventions are most effective when they are culturally tailored and grounded in behavioral change theory with active community input and systems-level coordination [95]. Community-wide interventions involving schools and local government, such as the Go-Golborne project in England, can reduce unhealthy behaviors such as SSB intake, fruit and vegetable consumption, and car travel to and from school [96]. Similarly, community-based interventions in Australia were noted to be more effective in children from low SES [97].

## 6. Home and Family Environment

The home and family environment represents a proximal context through which broader social, economic, and environmental determinants are transmitted to the child, shaping daily behaviors and physiological regulation relevant to obesity risk (Figure 1). The home and family environment strongly shape children’s dietary preferences, eating behaviors, physical activity patterns, sleep routines and emotional regulation. These influences are mediated through feeding practices, parenting styles, household routines, resources and exposure to stress or adverse experiences.

### 6.1. Feeding Practices and Parenting Styles

Authoritative parenting, characterized by responsiveness to a child’s hunger and satiety cues and high expectations, is protective against excessive weight gain [55,98,99]. Children raised in authoritative households tend to eat more nutritious foods and are more physically active compared to those from authoritarian, permissive or negligent homes [98,100,101,102,103]. In contrast, authoritarian, permissive/indulgent, and neglectful parenting styles are related to a heightened risk of childhood obesity, likely through reduced self-regulation of food intake and less structured home environments [99,100,101,102,103]. In a longitudinal study of 1602 children in the Québec Longitudinal Study of Child Development in which parenting behaviors at ages 6, 8, 10, and 12 were analyzed, responsive and lenient parenting styles were linked to higher BMI z-scores two years later [101].

Several randomized and longitudinal studies (e.g., INSIGHT, NOURISH, and THRIVE) show that interventions teaching responsive or positive feeding and parenting practices can modestly improve children’s eating behaviors and slightly reduce BMI z-scores, particularly when started early in life [104,105,106,107,108,109,110]. However, effects are generally small and not always sustained, and adding intensive parenting training to standard behavioral obesity treatment does not consistently yield additional weight benefits.

### 6.2. Adverse Childhood Experiences, Psychosocial Stress and Family Communication

In a systematic review including 21 studies examining how family functioning relates to childhood and adolescent obesity, twelve of seventeen observational studies found that children from families with poor communication, limited behavioral control, or frequent conflict were more likely to be overweight or have obesity [111]. Two intervention studies also showed that improving family functioning was linked to reductions in child weight [111].

Epidemiological evidence consistently links adverse childhood experiences (ACEs)—including abuse, neglect, household dysfunction, poverty, and exposure to violence—to greater odds of pediatric obesity in a dose–response relationship [37]. Meta-analyses show that higher cumulative ACE exposure is related to progressively higher odds of overweight and obesity in children and adolescents, with physical, sexual, emotional abuse and neglect conferring particularly elevated risk [33,37].

Chronic psychosocial stress related to ACEs may induce toxic stress, leading to dysregulation of neuroendocrine and metabolic pathways, while also promoting maladaptive coping behaviors such as emotional or binge eating, impulsive eating, reduced physical activity, and poor sleep hygiene [33]. At a biological level, chronic stress stimulates the hypothalamic–pituitary–adrenal (HPA) axis, leading to sustained cortisol exposure, which can increase appetite, favor energy-dense food preferences, and promote central fat accumulation [112,113]. Maternal stress during pregnancy can influence fetal metabolic programming, increasing later obesity risk phenomenon explored within the developmental regions of health and disease framework [112,113].

### 6.3. Sleep Routines

Inadequate sleep in infancy—particularly late bedtimes and frequent nighttime awakenings—is linked to decreased sleep duration and increased BMI later in childhood, whereas longer sleep duration has a protective effect [34,35,36]. In a meta-analysis including 57,848 children and adolescents, a remarkable relationship between sleep duration and obesity risk was identified [35]. Short sleep duration was related to a greater risk of obesity (adjusted RR = 1.57) and higher BMI z-scores, while long sleep duration appeared protective (RR = 0.83). The relationship between short sleep and obesity was strongest in school-aged children, followed by preschoolers and toddlers.

### 6.4. Cultural Beliefs and Norms

Cultural norms and parenting beliefs influence daily behaviors related to diet and activity. For instance, varying perceptions of ideal body size, feeding practices, and definitions of health can shape eating habits and physical activity levels within different communities [57,114]. Racial and ethnic differences in the prevalence of pediatric obesity likely reflect a complex interplay of biological, cultural, socioeconomic, and environmental factors rather than any single etiologic pathway. This complexity underscores the importance of prevention and treatment strategies grounded in a socioecological framework that are culturally responsive and attentive to socioeconomic and educational contexts [114]. Acculturation and globalization frequently contribute to the adoption of energy-dense, processed foods and more sedentary lifestyles, particularly among immigrant families adapting to Western environments. This transition, characterized by the replacement of traditional dietary patterns and physical activity routines with those common in the host culture, is linked to an elevated risk of obesity [57,115]. Immigrant cohort studies suggest that a longer duration of residence and greater acculturation to Western dietary and activity patterns are associated with higher obesity risk among children, highlighting the intergenerational nature of culturally mediated environmental exposure [57,115]. These observations underscore the importance of culturally tailored obesity prevention strategies, particularly for children from minority and immigrant backgrounds.

Together, these findings highlight that socioeconomic, cultural, and family environments interact bidirectionally across the course of life, and that parenting behaviors, household stress, and child health trajectories both shape and are shaped by broader structural conditions.

## 7. Digital and Media Environment

Screen media use has been connected to a higher risk of obesity, driven by behaviors like eating while watching, exposure to unhealthy food advertising, and poor sleep [38,39]. In a meta-analysis, ≥2 h/day of screen time was linked to 67% greater odds of overweight/obesity compared with <2 h/day (OR = 1.67; 95% CI: 1.48–1.88, *p* < 0.0001) [116]. Similarly, a large U.S. study reported that adolescents who watched television or played video games for ≥4 h/day had more than double the odds of overweight/obesity (aOR = 2.19; 95% CI: 1.73–2.77), and those using computers or handheld devices for ≥4 h/day had 53% greater odds (aOR = 1.53; 95% CI: 1.19–1.97) [18]. In a study of 23,183 Iranian students, screen time of ≥2 h per day was also linked to remarkably greater BMI z-scores (boys: 0.15 ± 1.12, girls: 0.17 ± 1.08, *p* < 0.001). However, interpretation of these associations is complicated by substantial heterogeneity in how “screen time” is defined and measured across studies, including differences in device type (television vs. mobile devices), context (educational vs. recreational use), self-report versus parent-report, and lack of distinction between concurrent behaviors such as eating or physical inactivity [117]. Additionally, although these associations are robust, concerns regarding reverse causality remain, as children with higher BMI may be more likely to engage in sedentary screen-based behaviors due to physical, social, or psychosocial constraints [118]. High screen time clusters with other obesity-related behaviors. Adolescents using other screen devices for ≥5 h/day had increased odds of inadequate sleep (aOR = 1.79; 95% CI: 1.54–2.08) and obesity (aOR = 1.78; 95% CI: 1.40–2.27) [119]. In addition, late bedtime combined with >30 min of screen use before sleep was related to substantially increased odds of overweight/obesity among toddlers (OR = 3.42; 95% CI: 1.41–8.26) and school-aged children (OR = 2.53; 95% CI: 1.10–5.03), alongside poorer sleep quality and shorter duration [120]. Longitudinal designs and isotemporal substitution analyses help to address, but do not fully eliminate, concerns of reverse causality by modeling the health impact of reallocating time between behaviors. In an isotemporal-substitution analyses using data from the Korean Children and Youth Panel Survey 2018, reallocating 1 h/day from screen time to sleep was linked to 31% decreased odds of obesity (OR = 0.69; 95% CI: 0.62–0.78), and reallocating to physical activity was linked to 25% decreased odds (OR = 0.75; 95% CI: 0.65–0.85) [56]. Replacing just 10 min of sedentary time with an equivalent amount of sleep and or physical activity could decrease overweight/obesity risk by 2.3–4.4% [121]. However, residual confounding and bidirectional relationships cannot be fully excluded, underscoring the need for cautious causal interpretation.

Interventions aimed at reducing screen time have demonstrated modest, short-term benefits in slowing weight gain and improving lifestyle [122]. Reduction in screen time in a randomized controlled trial of 70 children, aged 4–7, led to reductions in BMI z-score and energy intake compared with controls [123]. Furthermore, an 18-lesson “SMART” curriculum aimed at reducing TV, video, and gaming time in a school-based randomized controlled trial of children (grades 3–5) demonstrated lower BMI and improved physical activity compared to control schools [124]. Additionally, there is growing interest in using digital tools to encourage healthier habits [125,126]. In a 6-month randomized controlled trial in Sweden, a smartphone application designed to support parents in reinforcing healthy behaviors in their preschool-aged children was linked to decreased intakes of sugary drinks and treats, reduced screen time, and greater parental self-efficacy in promoting healthy lifestyle behaviors among children in the intervention group [127]. Emerging digital health intervention studies show promise in supporting behavior change but remain heterogeneous in design and outcome assessment.

Digital media exposure frequently clusters with shorter sleep duration, higher exposure to unhealthy food marketing, and decreased physical activity, underscoring the importance of considering time-use tradeoffs and co-exposures in epidemiologic analyses [35,36,57,114].

## 8. Early Life Factors

### 8.1. Developmental Origins of Health and Disease (DOHaD)

The Developmental Origins Of Health and Disease (DOHaD) hypothesis posits that exposures during the critical period of early development, particularly in the prenatal, periconceptional and early postnatal life, can “program” an individual’s risk for childhood obesity and related metabolic diseases through lasting changes in physiology and gene expression [40,41,42]. Prenatal factors such as high maternal prepregnancy BMI, gestational diabetes and excessive gestational weight gain are consistently associated with increased risk of childhood obesity [40]. However, effect sizes vary across populations, and these associations should be interpreted as probabilistic rather than deterministic. These effects are hypothesized to occur via epigenetic modifications such as DNA methylation, histone modification and microRNA regulation that persist beyond birth and may even be transmitted across generations [40,128,129]. Perinatal and early life factors associated with obesity include high birth weight and accelerated weight gain in infancy [130,131]. Notably, evidence linking high birth weight to later obesity demonstrates heterogeneity across cohorts, with effect estimates influenced by maternal metabolic status, gestational age, and postnatal growth patterns, and residual confounding cannot be fully excluded [130,131].

### 8.2. Early Nutrition, Microbiome and Antibiotics

There is robust evidence supporting a protective relationship between breastfeeding and the risk of pediatric obesity [132,133,134,135]. A systematic review demonstrated that continued or prolonged breastfeeding is associated with lower BMI trajectories from infancy through adolescence, with differences becoming apparent as early as 7 months of age and persisting up to 18 years [132]. Both exclusive and any breastfeeding were linked to more favorable BMI trajectories compared with formula or mixed feeding, although the magnitude of protection varies across studies and may be partially influenced by maternal, socioeconomic, and lifestyle factors [132,133,134].

Antibiotic use in infancy, especially repeated courses or exposure within the 1st 6–12 months, is linked to a modest but statistically significant increase in the risk of childhood overweight and obesity [58,136,137]. However, reported effect sizes are small and heterogeneous, and confounding by indication (e.g., underlying infections, healthcare utilization, or socioeconomic context) cannot be fully ruled out despite multivariable adjustment. The proposed mechanism is hypothesised to involve antibiotic-induced dysbiosis of the gut microbiome. The infant gut microbiome is established during the first years of life and has an important role in energy balance and metabolic programming. Antibiotics can reduce microbial diversity and alter the availability of key taxa, such as Bifidobacterium, Eubacterium halli, and others, which are negatively correlated with adiposity [43,44]. Evidence linking specific microbiome alterations to sustained obesity risk in humans remains preliminary. The American Academy of Pediatrics recommends judicious use of antibiotics in infancy, favoring narrow-spectrum agents when possible.

## 9. Endocrine Disruptors and Chemical Environmental Exposures

Multiple epidemiological and mechanistic studies indicate that exposure to bisphenol A (BPA) and phthalates is related to greater adiposity and obesity risk in the pediatric population, with evidence suggesting vulnerability during both prenatal and postnatal periods [59,138,139,140,141]. Effect sizes are generally small and vary across studies, reflecting differences in exposure timing, sex, and outcome definitions. In a systematic review of 13 studies with 173–1124 mother–child pairs, eight studies concluded that prenatal BPA exposure was related to heightened obesity in offspring [139]. Parabens and polyfluoroalkyl substances (PFAS) have also been associated with adiposity-related outcomes, although findings are heterogeneous, with several cohorts reporting sex-specific effects or null associations, particularly in adolescents [140,142]. Heavy metals such as lead and cadmium and organophosphates are recognized as potential obesogens, particularly through epigenetic and metabolic disruption [45]. However, exposure misclassification and residual confounding, particularly by socioeconomic and co-exposure factors, remain important limitations across studies. These chemicals can disrupt endocrine signalling during critical developmental windows, including prenatal, neonatal and early childhood periods, leading to altered adipogenesis, metabolic programming and increased obesity risk [45,46]. The American Academy of Pediatrics highlights the importance of minimizing exposure to these endocrine disruptors to reduce obesity risk in children [5].

Childhood obesity has also been consistently but modestly associated with exposure to ambient air pollutants, particularly particulate matter (PM_1_, PM_2.5_, PM_10_), nitrogen oxides (NO2, NO_x_) and ultrafine particles, with evidence supporting both higher BMI and higher odds of overweight/obesity in exposed children [60,143]. A recent systematic review by Malacarne et al. showed strong evidence indicating that traffic-related pollutants—including NO_2_ and NO_x_—are associated with childhood obesity outcomes (*p* < 0.001) [17]. In a systematic review and meta-analysis of 15 studies conducted across seven countries, long-term exposure to air pollutants—particularly PM_1_, PM_2.5_, PM_10_ and NO_2_—was significantly related to an increased risk of childhood obesity [143] (Table 3). Additionally, in a meta-analysis of ten European birth cohorts, prenatal exposure to PM_2.5_ was linked to a 23% higher risk of childhood overweight or obesity (95% CI: 1.05–1.37), and higher BMI z-scores and increased overweight/obesity risk observed at ages 9–12 years [144]. Both prenatal and postnatal exposures are relevant, with some evidence suggesting that prenatal PM_2.5_ exposure increases obesity risk later in childhood [144]. Associations varied by exposure window and cohort, and causal inference remains limited by potential residual confounding and spatial exposure misclassification. Mechanistically, air pollution is hypothesized to promote obesity through pathways involving inflammation, oxidative stress, disruption of gut microbiota and metabolic imbalance [145]. Overall, while the literature supports a relationship between environmental pollutants and pediatric obesity, effect sizes are modest, and uncertainties remain, particularly for certain PFAS compounds and metals, underscoring the need for further longitudinal studies with improved exposure assessment.

## 10. Implications for Prevention and Intervention

**Figure 2:**
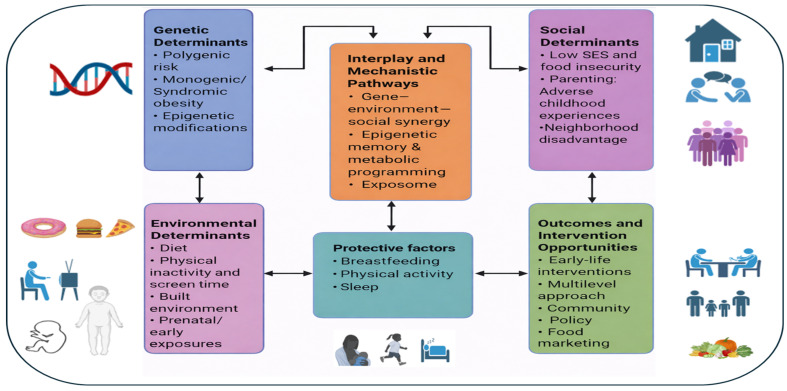
Summary of the key interrelated determinants contributing to childhood obesity and the multilevel interventions required to effectively address these factors.

The complex interplay among genetic, environmental, and social factors underscores the necessity for personalized, multilevel and context-sensitive approaches to pediatric obesity prevention (Figure 2). Effective strategies should operate across individual, family, community, and policy levels. Multi-sectoral, community-wide interventions—such as the Amsterdam Healthy Weight Program and similar US city initiatives—have combined urban planning, school-based changes, food environment regulation, and community engagement [146]. The Amsterdam Healthy Weight Program showed a notable reduction in obesity prevalence among the most deprived children aged 0–18 years, with rates falling from 8% to 6% between 2012 and 2015. The overall prevalence of overweight and obesity in this age group declined from 21% to 18.5% during the same period [146,147]. However, the magnitude and sustainability of these effects vary across subpopulations and implementation contexts, highlighting the importance of local adaptation and sustained investment.

In the United States, comprehensive neighborhood-focused strategies implemented in cities such as New York City and Philadelphia have been associated with modest declines in obesity prevalence among school-aged children [146]. Similar comprehensive strategies have also shown success in lowering childhood obesity (0–5 years) in Leeds, UK, [148], and in four USA projects [149]. In the USA, strategies in New York City and Philadelphia targeted students in grades K-8 (i.e., age 5–14 years) [149]. In New York City, obesity in this age group over the period 2006–07 to 2010–11 declined from 21.9% to 20.7%, representing a relative decline of 5.5% (*p* < 0.001). Notably, the effectiveness of such interventions often differs by socioeconomic context, with greater benefits observed in settings where structural barriers to healthy behaviors are simultaneously addressed.

Despite their promise, population-level policy interventions—including SSB taxes and local regulatory approaches—are not uniformly effective and may yield heterogeneous effects across income and racial/ethnic groups. Evidence suggests that while SSB taxes can reduce purchases and consumption, their impact on obesity outcomes is modest and may be attenuated in communities facing persistent food insecurity or limited access to healthy alternatives [146,150]. Moreover, public health policies may carry unintended consequences, including the risk of stigmatization or the potential to exacerbate existing health inequities if not carefully designed and implemented with equity considerations.

Collectively, these findings underscore that policy and community interventions are most effective when embedded within broader equity-focused strategies that address underlying social determinants, engage affected communities, and anticipate unintended effects. Future prevention efforts should prioritize evaluation of long-term outcomes, differential impacts across populations, and strategies that minimize stigma while maximizing health equity.

## 11. Gaps in Current Research and Future Directions

There remains a limited understanding of which intervention components are most effective for specific subgroups, as many studies fail to account for the heterogeneity of risk factors and social determinants such as poverty and ACEs. There is a critical need for rigorous environmental intervention studies that move beyond observational associations to evaluate the causal and longitudinal impacts of alterations in food, built, and digital environments on childhood obesity outcomes. Long-term, longitudinal assessments of digital exposures, including evolving patterns of screen use and digital marketing, are also lacking, limiting understanding of sustained impacts across developmental stages.

Few longitudinal or interventional studies examine obesogenic exposures (e.g., air pollution, urban design metrics, and food deserts) in many non-Western regions, particularly in sub-Saharan Africa, South Asia, and parts of the Middle East and Southeast Asia, and the available evidence remains sparse, with most causal evidence derived from high-income countries [12,15]. Additionally, standardized exposure measurement and culturally adapted definitions (e.g., of walkability or food swamps) are often lacking, limiting cross-regional comparisons [151]. There is a critical need for regionally contextualized studies to characterize environmental determinants of pediatric obesity across diverse sociocultural settings. In addition, translational research informed by the DOHaD framework is needed to bridge early life biological insights with scalable prevention strategies across the life course.

## 12. Conclusions

Several environmental factors are associated with childhood obesity. Associations with food environments, screen time, and sleep are supported by consistent findings from longitudinal cohorts and meta-analyses, whereas evidence for endocrine-disrupting chemicals and air pollution remains emerging, heterogeneous, and largely observational [13,78]. These factors disproportionately cluster within socioeconomically disadvantaged communities and among racial and ethnic minority populations. Much of the current evidence base remains observational, with residual confounding and bidirectional relationships limiting causal inference for several environmental exposures. Future studies should prioritize improved exposure assessment, longitudinal and quasi-experimental designs, and equity-focused intervention trials to better delineate causal pathways and inform precision prevention strategies.

**Table 3:** Characteristics of key studies included in the review according to study design, country, and results.

Environmental Domain	First Author (Year)	Country	Study Design	Key Exposure(s)	Key Outcome(s)	Result
**Food Environment**	Al-Hazzaa (2012) [68]	Saudi Arabia	Cross-sectional	SSBs, infrequent breakfast and vegetable intake	Overweight/obesity	Lower consumption of SSB (<3 days/week) was linked to greater odds of overweight/obesity: for BMI-based overweight/obesity, aOR = 1.32 (95% CI: 1.08–1.62). For WHtR-based abdominal obesity, aOR = 1.42 (95% CI: 1.16–1.75).
	Du (2024) [62]	US	Cohort	UPF consumption	BMI	Each 10% increase in UPF intake was linked to a yearly BMI increase of 0.01 (95% CI: 0.003–0.03) and a cumulative increase of 0.07 (95% CI: 0.01–0.13) over five years.
	Costa (2021) [63]	Brazil	Cohort	UPF consumption	FMI	An increase of 100 g/day in UPF intake was associated with a 0.14 kg/m^2^ increase in FMI.
	Petridi (2024) [64]	Multi-country	Systematic review	UPF consumption	Overweight/obesity and cardiometabolic outcomes	Most studies (14/17, 82%) showed that elevated UPF consumption was related to greater prevalence of overweight/obesity and cardiometabolic comorbidities.
	Rossi (2019) [152]	Brazil	Cross-sectional	Food access, social assistance	BMI	Among low-income families: living> 11 minutes’ walk from parks/playgrounds was linked to higher BMI (β = 0.53; 95% CI = 0.33–0.73) [1]. Among high-income families: increased distance from home to football pitches was linked to decreased BMI (β = −0.49; 95% CI = −0.69; −0.29.
	Pineda (2024) [78]	Multicountry	Systematic review and meta-analysis	Density of fast-food outlets and supermarkets Availability of fresh fruit and vegetable outlets	Odds of obesity	Fast-food outlet proximity was associated with greater obesity odds (OR ≈ 1.15; 95% CI: 1.02–1.30). Fresh fruit and vegetable outlet density was associated with lower obesity risk (OR ≈ 0.90; 95% CI: 0.82–0.98). Supermarket proximity was inversely linked to obesity (OR ≈ 0.93; 95% CI: 0.90–0.96).
**Built Environment and Physical Activity**	Aris (2022) [87]	USA (ECHO)	Multi-cohort	COI, SVI	BMI	Children living in areas with higher COI scores had decreased mean BMI and a reduced likelihood of obesity over time compared with those in areas with very low COI, with a mean BMI difference of β = –2.58 (95% CI: –2.95 to –2.21); obesity risk: RR = 0.21 (95% CI, 0.12–0.34).
	Pereira (2019) [88]	Portugal	Ecological	Green space	Obesity	Living in newer buildings with parking and urban green areas was protective against pediatric obesity (OR 0.44, 95% CI 0.25–0.80).
	Putra (2022) [27]	Australia	Longitudinal cohort	Perceived safe neighborhoods Improved green spaces Greater access to shopping facilities High traffic exposure	Increase in BMI (moderate, high and extreme)	Very safe vs safe lower risk (adjusted RRR for rise in BMI 0.83, 95% CI 0.76–0.90). Green space, high quality vs low quality, decreased risk of extreme increase (RRR 0.60, 95% CI 0.43–0.84) Higher risks of extreme BMI gain (RRRs 1.46 to 1.64). High traffic vs low traffic: extreme increase in BMI (RRR 1.35, 95% CI 1.11–1.64).
**Socioeconomic and Community Context**	Anderson (2022) [90]	Canada	Cross-sectional analysis	Family and neighborhood level income, neighborhood deprivation	BMI, BMI-z score	Decreased family income, OR = 4.69, 95% CI 2.65–8.29), low neighborhood income, OR = 2.18, 95% CI 1.33–3.58), and high neighborhood deprivation, OR = 2.45, 95% CI 1.52–3.95) were each independently linked to higher odds of pediatric obesity. After adjustment for family income, the associations for neighborhood income (OR = 1.39, 95% CI 0.82–2.34) and deprivation (OR = 1.56, 95% CI 0.94–2.58) were attenuated, suggesting that family-level income was the stronger predictor.
	Buoncristiano (2021) [92]	Multi-country	Cross-sectional	Parental education, employment and family-perceived wealth	Overweight/obesity	High-income countries: lower parental education was linked to greater obesity prevalence (OR: 1.78; 95% CI: 1.36–2.32). Middle-income countries: lower parental education linked to lower obesity prevalence (OR: 0.46; 95% CI: 0.34–0.62. Family-perceived wealth showed similar patterns to parental education.
	St. Pierre (2022) [32]	US	Systematic review	Household food insecurity	BMI, BMI-z score, obesity	Food insecurity was linked to greater weight gain in early childhood, for girls, and for children experiencing food insecurity at multiple time points.
	Jacobs (2025) [97]	Australia	Meta-analysis	CBI, SEP	BMI z-score, weight related behaviors	Intervention effect was higher in low compared to high-SEP students (intervention effect difference = −0.10 [95% CI −0.18, −0.02]), suggesting that obesity prevention CBIs may reduce rather than widen health inequities.
**Home and Family Environment**	Shloim (2015) [98]	Multi-country	Systematic review	Parenting, feeding styles and practices	BMI, change in weight, obesity	Uninvolved, indulgent, or highly protective parenting was related to greater child BMI, whereas authoritative parenting was linked to healthy BMI. Indulgent feeding was consistently related to risk of obesity. Restriction and pressure to eat were linked to BMI. However, the feeding style was responsive to the child—restriction was used with children with elevated BMI and pressure to eat with children with decreased BMI.
	Paul (2018) [104]	US	Randomized clinical trial	RP educational intervention	BMI z-score at age 3 years	At age 3 years, the RP group had a significantly lower BMI z-score (0.77 vs. 0.94; difference: −0.17, 95% CI: −0.33 to −0.01; *p* = 0.04) and lower overweight/obesity prevalence (23.9% vs. 36.5%; OR: 0.54, 95% CI: 0.31–0.95; *p* = 0.03) compared to controls.
	Lamichhane (2020) [112]	Denmark	Systematic review	Prenatal psychosocial stress, adverse life events, stress hormones	Obesity, BMI	8 of the 15 studies found a direct association between exposure to stress in fetal life and obesity measures in offspring. The direct association was most consistent in maternal exposure to natural disasters, suggesting that more severe or objective stressors may have stronger effects.
	Danial (2023) [34]	Sweden	Prospective cohort	Sleep dimensions	BMI z-score, overweight, obesity	Sleep duration early in life was negatively linked to BMI z-scores later in childhood (adjusted β = −0.09, 95% CI: −0.15 to −0.03, *p* = 0.005). Later bedtime was strongly linked to shorter sleep duration (β = −0.544, *p* < 0.0001).
	Mcleod (2016) [115]	USA	Systematic review	Acculturation	BMI, BMI z-score, overweight/obesity prevalence	Across 29 reviewed studies, the association between acculturation and obesity among Latino children was inconsistent, with mixed findings and variable strength and direction of associations.
**Digital and Media Environment**	Fang (2019) [116]	China	Systematic review and met analysis	Screen time	Overweight, Obesity	≥2 h/day of screen time was associated with 67% increased odds of overweight/obesity compared with <2 h/day (OR = 1.67; 95% CI: 1.48–1.88).
	Djalalinia (2017) [117]	Iran	Cross-sectional	Screen time, physical activity	BMI z-score, WC, hip circumference, overweight and obesity prevalence	Students in the “Low PA & High ST” group had the highest levels of: BMI z-scores (boys: 0.15 ± 1.12, girls: 0.17 ± 1.08), WC (boys: 69.93 ± 13.89 cm, girls: 67.30 ± 11.26 cm), hip circumference (boys: 82.41 ± 13.90 cm, girls: 84.05 ± 13.90 cm), prevalence of overweight (boys: 15.32%, girls: 14.04%), (*p* < 0.001 for all comparisons).
**Early Life Factors (DOHaD)**	Dewey (2021) [133]	USA	Systematic review	Breastfeeding	Overweight/obesity	Ever consuming human milk, compared with never consuming it, is linked to a lower risk of overweight and obesity at ages 2 years and older, especially if the duration of human milk consumption exceeds 6 months.
	Saha (2022) [135]	India	Cross sectional	Child characteristics, maternal and household factors	Overweight/obesity	Factors related to increased risk of overweight/obesity: male sex: ARR 1.08, age 0–11 months: ARR3.77 (highest risk age group), low birth rank: ARR 1.24. Maternal obesity: ARR 1.81, maternal marriage after age 18: ARR 1.15, scheduled tribe family: ARR 1.46, higher dietary diversity (7–9 food items): ARR 1.22. Factors associated with decreased risk (protective factors): breastfeeding: ARR 0.85, Muslim families: ARR 0.87.
**Endocrine Disruptors and Chemical Environmental Exposures**	Jaskulak (2025) [140]	Multicountry	Systematic review	Phthalates, parabens, bisphenols, PFAS, organochlorine pesticides	Obesity and metabolic outcomes	Consistent associations were found between exposure to phthalates, parabens, and bisphenols and obesity or metabolic outcomes in children and women. Results for PFAS and organochlorine pesticides were more variable, especially in adolescents and adults.
	Malacarne (2022) [17]	Multi-country	Systematic review	NO_2_ and NO_x_	Obesity	Strong association was found between air pollution (nitrogen dioxide and nitrogen oxides exposure) and pediatric obesity *p* < 0.001.
	Huang (2022) [143]	Multi-country	Systematic review and meta-analysis	PM_1_, PM_2.5_, PM_10_ and NO_2_	Obesity	Air pollutants were significantly associated with childhood obesity and weight gain. For obesity risk (odds ratios per 10 μg/m^3^ increment): PM_10_: OR = 1.12 (95% CI: 1.06, 1.18), PM_2.5_: OR = 1.28 (95% CI: 1.13, 1.45), PM_1_: OR = 1.41 (95% CI: 1.30, 1.53), NO_2_: OR = 1.11 (95% CI: 1.06, 1.18). For BMI status (regression coefficients β per 10 μg/m^3^ increment): PM_10_: β = 0.08 kg/m^2^ (95% CI: 0.03, 0.12), PM_2.5_: β = 0.11 kg/m^2^ (95% CI: 0.05, 0.17), NO_2_: β = 0.03 kg/m^2^ (95% CI: 0.01, 0.04).
	Warkentin (2025) [144]	10 European countries	Meta-analysis	Pre- and postnatal exposure to PM_2.5_ and NO_2_	Obesity risk	Prenatal PM_2.5_ exposure: related to 23% higher odds of overweight/obesity across childhood (OR 1.23, 95% CI: 1.05, 1.37). NO_2_ exposure: no robust associations were found with BMI z-score or overweight/obesity risk for either prenatal or postnatal exposure. Postnatal PM_2.5_ and NO_2_: No evidence supported an effect of postnatal air pollution exposure on childhood obesity outcomes

Abbreviations: SSBs, sugar-sweetened beverages; BMI, body mass index; BMI z-score, age- and sex-standardized body mass index; aOR, adjusted odds ratio; OR, odds ratio; RR, risk ratio; RRR, relative risk ratio; ARR, adjusted risk ratio; CI, confidence interval; β, regression coefficient; *p*, *p*-value; WHtR, waist-to-height ratio; WC, waist circumference; UPF, ultra-processed foods; FMI, fat mass index; COI, Child Opportunity Index; SVI, Social Vulnerability Index; ECHO, Environmental Influences on Child Health Outcomes consortium; SEP, socioeconomic position; CBI, community-based intervention; PA, physical activity; ST, screen time; RP, responsive parenting; DOHaD, Developmental Origins of Health and Disease; PFAS, per- and polyfluoroalkyl substances; NO_2_, nitrogen dioxide; NO_x_, nitrogen oxides; PM_1_, particulate matter ≤1 μm in aerodynamic diameter; PM_2.5_, particulate matter ≤2.5 μm in aerodynamic diameter; PM_10_, particulate matter ≤10 μm in aerodynamic diameter; US, United States.

## Figures and Tables

**Table 1:** Key environmental drivers of childhood obesity.

Environmental Domain	Specific Drivers	Mechanisms Linking to Obesity	References
Food Environment	UPF availability	Increased caloric intake	[14,19]
	Low cost of calorie-dense foods	Preference for hyper-palatable foods	
	Food swamps	Impulse-driven snacking	[20,21]
	Child-targeted marketing	Reinforced cravings	[22,23]
	School and childcare nutrition quality		
Built Environment	Low walkability	Reduced moderate to vigorous physical activity	[17,24,25]
	Lack of sidewalks/bike lanes	Sedentary behavior	[25,26,27,28]
	Limited parks	Less outdoor play	
	High crime and traffic danger		
	Limited school PE		
Socioeconomic Context	Poverty and food insecurity	Cyclical overeating	[29]
	Housing instability	Cortisol-driven adiposity	[30,31]
	Neighborhood deprivation		
	Psychosocial stress	Reliance on cheap calorie-dense foods	[5,32]
Home and Family	Unhealthy home food availability	Impaired satiety	[33]
	Nonresponsive feeding	Emotional eating	
	Irregular routines	Snacking during screens	
	Sleep disruption		[34,35,36]
	ACEs		[37]
Digital Environment	High screen time	Sedentary behavior	[38,39]
	Digital marketing	Mindless eating	
	Influencer promotions	Algorithmic exposure to unhealthy foods	
	Video games		
Early Life and Chemical	Maternal obesity and gestational diabetes	Metabolic programming	[40,41,42]
	Early antibiotics	Microbiome-mediated risk	[43,44]
	Formula feeding		
	EDCs: BPA, phthalates, PFAS	Adipogenesis via PPARγ	[45,46]

Abbreviations: UPF, ultra-processed foods; PE, physical education; ACEs, adverse childhood experiences; EDCs, endocrine-disrupting chemicals; BPA, bisphenol A; PFAS, per- and polyfluoroalkyl substances; PPARγ, peroxisome proliferator-activated receptor gamma.

**Table 2:** Summary of key effect estimates of pivotal studies reported in the review.

Domain	Setting	Exposure	Outcome	Effect
Food Environment	2011–2016 NHANES [47]	Highest consumption of UPF versus lowest consumption of UPF	Total, abdominal, and visceral overweight/obesity	Total overweight/obesity: OR 1.45, 95% CI 1.03–2.06, *p* = 0.040 Abdominal overweight/obesity OR 1.52, 95% CI 1.06–2.18, *p* = 0.026 Visceral overweight/obesity; OR 1.63, 95% CI 1.19–2.24, *p* = 0.005
Food Environment	9025 British children followed from 7 to 24 years of age [48]	Highest vs lowest quintile of UPF consumption	Trajectory of BMI, FMI, weight and WC	Trajectories of BMI increased by an additional 0.06/year (95% CI, 0.04–0.08); FMI, by an additional 0.03 (95% CI, 0.01–0.05) per year; weight
Food Environment	Systematic review and meta-analysis [49]	Serving of SSB per day	BMI	Each additional serving of SSB/day was associated with a 0.07-kg/m^2^ (95% CI: 0.04 kg/m^2^, 0.10 kg/m^2)^ compared to controls
Food Environment	Meta-analysis [50]	High intake of SSB	Odds of overweight/obesity	Higher intake of sugar-sweetened beverages increased the odds of overweight/obesity OR 1.20 (95% CI, 1.09–1.33, *p* < 0.05)
Built Environment	US Environmental Influences on Child Health Outcomes consortium 1994–2023 [51]	Low income, low food access vs non-low-income low food access neighborhood during pregnancy or early childhood	Obesity Risk	Higher obesity risk at 5 years (RR, 1.37; 95% CI, 1.21–1.55) and at 10 years (RR, 1.71; 95% CI, 1.37–2.12), and 15 years (RR, 2.08; 95% CI, 1.53–2.83)
Built Environment	9-year ECLS-K, followed 1998–2007 cohort [52]	Street intersection density, residential density	Obesity Risk	increased intersection density had decreased obesity odds in 2007 (OR = 0.79 [95% CI = 0.66–0.94]), particularly in girls (OR = 0.68 [95% CI = 0.52–0.88]) higher residential density in 1998 showed lower obesity risk (OR = 0.54 [95% CI = 0.30–0.98]) and risk of overweight (OR = 0.54 [95% CI = 0.30–0.95]) in 2007
Built Environment	Urban, pediatric integrated delivery system, (N = 51,873, ages 6–19 years, 77% African American) [25]	Neighborhood greenness and perceived safety	BMI z score	Increases in neighborhood green spaces and perceived safety were related to decline in BMI z-score (mean change in BMI z-score for 1-SD increase for both: −0.012; 95% CI= (−0.018, −0.007)
Socioeconomic and Community Context	Seven population-representative child cohorts from six HICs [53]	Maternal education and household income	Risk for obesity	Risk of obesity for low maternal education (pooled RR: 2.99, 95% CI: 2.07, 4.31) and low household income (pooled RR: 2.69, 95% CI: 1.68, 4.30)
Socioeconomic and Community Context	2017–2018 NHANES [54]	Food insecurity	Risk for obesity and abdominal obesity	Risk of obesity (aOR: 1.59 [95% CI: 1.19–2.13]) and abdominal obesity (aOR: 1.56 [95% CI: 1.19–2.03]
Home and Family Environment	1994–2008 cross-sectional samples of the NLSCY, a nationally representative survey of Canadian youth [55]	Parenting Style	Risk of obesity	Compared to authoritative parenting, preschool- and school-age children with authoritarian parents had 35% (95% CI: 1.2–1.5) and 41% (CI: 1.1–1.8) greater odds of obesity, respectively
Home and Family Environment	Minnesota Student Survey, n = 105,759 public school students [37]	ACEs	Risk of obesity and severe obesity Referent no ACE	1 ACE obesity OR 1.38 (1.30–1.47) and severe obesity 1.49 (1.37–1.63) 6 ACEs obesity OR 2.03 (1.33–3.1) and severe obesity 4.24 (2.71–6.65)
Home and Family Environment	Meta-analysis of 33 articles (57,848 children) [35]	Sleep Routines	Risk of obesity	Decreased sleep duration linked to greater risk of obesity (adjusted RR = 1.57, 95% CI: 1.36 to 1.81, *p* < 0.001)
Digital and Media Environment	5180 adolescents, isotemporal substitution using data from the Korean Children and Youth Panel Survey 2018 [56]	Screen time and non-screen time activities	Odds of obesity	Prolonged smartphone use (≥180 vs. <60 min/day) was associated with 2.75 times higher odds of obesity (OR = 2.75; 95% CI: 2.06, 3.68). TV watching (≥120 vs. <60 min/day) was positively associated with obesity among 4th grade students (OR = 2.09; 95% CI: 1.51, 2.89) but not among 7th grade students (OR = 0.97; 95% CI: 0.63, 1.49). Replacing 1 h of screen time with any non-screen activity was related to lower obesity prevalence: physical activity (OR = 0.75; 95% CI: 0.65, 0.85), sleeping (OR = 0.69; 95% CI: 0.62, 0.78), hanging out with friends (OR = 0.80; 95% CI: 0.71, 0.89), reading (OR = 0.82; 95% CI: 0.69, 0.97), studying (OR = 0.84; 95% CI: 0.78, 0.90), and chatting with parents (OR = 0.89; 95% CI: 0.88, 0.98)
Early Life factors	Case–control of 509 preschool children randomly selected from Tehran [57]	Early life modifiable risk factors	Overweight/Obesity	Gestational diabetes had the highest predicted probability of childhood obesity (OR = 4.36; 95% CI: 1.94–9.80). Introduction of solid food before 4 months of age increased the risk of obesity by 2.98 times (95% CI: 1.77–4.97). Maternal overweight and obesity were associated with 2.72 times greater odds (95% CI: 1.60–4.60), maternal smoking with 2.21 times greater odds (95% CI: 1.18–4.11), and excessive gestational weight gain with 1.89 times higher odds (95% CI: 1.23–2.91). Paternal smoking and increased birth weight increased the risk by >1.8 times and >1.5 times, respectively
Early Life factors	747 mother–child pairs recruited during pregnancy and followed through childhood, (4 and 6 years of age [58]	Prenatal and postnatal antibiotic	BMI, WC, cardiometabolic risk factors	Prenatal exposure to antibiotics was associated with a 2-fold increase in the risk for obesity (RR = 2.09, 95% CI: 1.58–2.76) and abdominal obesity (RR = 2.56, 95% CI: 1.89–3.47) at 6 years. Postnatal exposure to antibiotics was associated with increased weight (β = 0.25, 95% CI: 0.06–0.44) and BMI (β = 0.23, 95% CI: 0.003–0.45)
EDCs	Systematic review and meta-analysis [59]	Bisphenol A and 2,5-dichlorophenol	Childhood obesity and measures of body fat	Association between exposure to bisphenol A and overweight (OR 1.254, 95% CI 1.005 to 1.564), obesity (OR 1.503, 95% CI 1.273 to 1.774) and increased waist circumference (OR 1.503, 95% CI 1.267 to 1.783) in adults, and between exposure to 2,5-dichlorophenol and obesity in children (OR 1.8, 95% CI 1.1018 to 3.184)
EDCs	Meta-analysis of 27 studies [60]	PM_10_ (per 10 µg/m^3^)	Risk of overweight/obesity	Pooled OR 1.11 (1.06, 1.17) per 10 μg/m^3^ increment
EDCs	Meta-analysis of 27 studies [60]	PM_1_ (per 10 µg/m^3^)	Risk of overweight/obesity	Pooled OR (95% CI) of 1.23 (1.09, 1.40), per 10 μg/m^3^ increment
	Meta-analysis of 27 studies [60]	PM_2.5_ (per 10 µg/m^3^)	Risk of overweight/obesity	Pooled OR 1.18 (1.10, 1.28) per 10 μg/m^3^ increment

Abbreviations: NHANES, National Health and Nutrition Examination Survey; UPF, ultra-processed foods; OR, odds ratio; CI, confidence interval; BMI, body mass index; FMI, fat mass index; WC, waist circumference; SSB, sugar-sweetened beverages; RR, risk ratio; ECLS-K, Early Childhood Longitudinal Study–Kindergarten Cohort; SD, standard deviation; HIC, high-income countries; aOR, adjusted odds ratio; NLSCY, National Longitudinal Survey of Children and Youth; ACEs, adverse childhood experiences; TV, television; PM_10_, particulate matter ≤10 μm in aerodynamic diameter; PM_1_, particulate matter ≤1 μm in aerodynamic diameter; PM_2.5_, particulate matter ≤2.5 μm in aerodynamic diameter; EDCs, endocrine-disrupting chemicals.

## Data Availability

No new data was generated or analyzed in support of this review.

## Appendix A

**Table A1:** Literature search strategy by database.

Database	Search Dates	Publication Date Range	Search Strategy (Boolean/Fields)	Screening Procedure
**PubMed**	20 July 2025–30 October 2025	1 January 1990–1 January 2025	(“Pediatric Obesity” [MeSH] OR “Childhood Obesity” [MeSH] OR pediatric obesity OR childhood obesity OR pediatric overweight) AND (“Environment” [MeSH] OR environmental exposure* OR built environment OR food environment OR endocrine disruptor* OR air pollution OR screen time OR socioeconomic factor*) AND (“Epidemiology” [MeSH] OR epidemiology OR prevalence OR risk)	Titles and abstracts screened for relevance; full texts reviewed when eligibility was unclear
**Web of Science**	20 July 2025–15 December 2025	1 January 1990–1 January 2025	TS = (pediatric obesity OR childhood obesity OR pediatric overweight) AND TS = (environment* OR built environment OR food environment OR endocrine disruptor* OR air pollution OR screen time OR socioeconomic) AND TS = (epidemiology OR prevalence OR risk)	Topic-based screening of titles and abstracts followed by full-text review
**Google Scholar**	20 July 2025–30 October 2025	1 January 1990–1 January 2025	Predefined keyword combinations aligned with database searches (e.g., pediatric obesity AND built environment; pediatric obesity AND food environment; pediatric obesity AND endocrine disruptors)	Results sorted by relevance; first 200 records per search string screened; titles and abstracts reviewed with full-text retrieval as needed

Abbreviations: MeSH, Medical Subject Headings; TS, topic search field.
